# JAK-STAT in lymphoproliferative disorders

**DOI:** 10.18632/oncoscience.189

**Published:** 2015-08-11

**Authors:** Raul Rabadan, Giorgio Inghirami

**Affiliations:** Department of Pathology and Laboratory Medicine, Weill Cornell Medical College, New York, New York, USA

**Keywords:** lymphomageneis, T-cell lymphoma, mutations, STAT3 signaling

In the early 1990's several groups were searching for the molecular basis of the signal transduction triggered by the engagement of plasma membrane receptors. The laboratories of Darnell, Kerr and Stark were first to identify proteins, which acted as intermediaries in interferon (IFN) signaling, known as the signal transducer and activator of transcription (STAT). At the same time, the work of Pellegrini's, Ihle's and Carter-Su's groups demonstrated that non-receptor tyrosine kinases, previously described by John Krolewski and Andrew Wilks, called Janus Kinases (JAK), played a role in cytokine receptor signaling. We have since learned that multiple negative regulators, mainly tyrosine phosphatase (SHP), Protein Inhibitors Against Stats (PIAS), and Suppressor Of Cytokine Signaling (SOCS) proteins, modulate and eventually extinguish JAK-STAT signaling [1].

In invertebrates a single JAK-STAT module controls anti-viral and anti-bacterial responses, leukocyte-like hemocyte generation, cell fate determination, brain development, cardiogenesis, as well as intestinal stem cells. The increase of JAK-STAT pathway components has coincided with the emergence of adaptive immunity and the expansion and diversification of cytokine receptors. As ligands bind to cognate receptors, they trigger the recruitment and activation of JAKs. Activated JAKs can then phosphorylate the receptor favoring the STAT docking and ultimately their activation via tyrosine phosphorylation. Phospho-STAT dimers accumulate in the cell nucleus, bind to enhancer elements and regulate gene expression. In parallel, JAKs may fire other downstream signaling cascades (MAP kinase and PI-3-kinase/AKT pathways), or within the nucleus by phosphorylating DNA regulatory proteins (histone H3 and methyltransferase) modulate gene expression and the epigenetic program of cells [2].

There is comprehensive evidence that abnormal JAK/STAT signals can lead to immunodeficiencies, a spectrum of cytokine mediated inflammatory diseases and cancer. Hyperactivation of STAT signaling is common in hematopoietic disorders through several different mechanisms [2-3]. JAK2 amplification, loss of SOCS1 and phosphatases, as well as somatic mutations of STAT3 and STAT6 are seen in mediastinal B-cell, grey zone, Hodgkin and Diffuse Large B-cell lymphomas. Activating mutations of JAK1-3 and STAT3-5 were also found in a subset of NK/T-cell, non-hepatosplenic gamma-delta T-cell lymphoma and T-cell prolymphocytic leukemia. Moreover, the constitutive activation of STAT is also observed in cells carrying tyrosine kinase fusions. This is epitomized in Anaplastic Large Cell Lymphomas (ALCL) carrying Anaplastic Lymphoma Kinase (ALK) fusions. In these settings, STAT3 inhibition inevitably leads to cell cycle arrest followed by apoptosis [4-5].

Searching for genomic defects responsible for the transformation and the maintenance of the neoplastic phenotype of ALK-ALCL, our groups have used massive genomic sequencing. These studies demonstrated the presence of recurrent activating mutations of JAK1 and/or STAT3 and novel tyrosine kinase fusions. JAK1 and STAT3 mutants result in a hyperactivated STAT3, which sustains cell transformation, and whose pharmacological ablation produces tumor cell growth inhibition. Interestingly, we found that ~30% of systemic pSTAT3 positive ALK-ALCL carry both JAK1 and STAT3 mutations that work synergistically [5]. Further analyses showed that in single JAK1 or STAT3 mutants, non-sense mutations and genomic loss of negative regulators (PTPRC/D) can be detected, suggesting that convergent mutations on the same pathway might be selected. However, the association of two mutations that are convergent in genes coding for two interplaying proteins is unexpected. The probability of mutations at a particular site is small, when randomly distributed along the genome, and conditionally independent of previous mutations. Thus the probability of double mutations is theoretically the square of the probability of a single mutation. The strong association between JAK/STAT mutations argues for a set of complementary and overlapping explanations: 1) a significant clonal expansion of single mutant fostering the emergency of susceptible tumor cells, 2) strong selection in double mutants, 3) differential off-pathway events of different mutations, or 4) preferential mutational hotspots. These hypotheses offer fascinating possibilities, including the identification of early clones that mark disease progression. Currently, our groups are trying to create a model for clonal evolution in ALCL.

**Figure 1:**
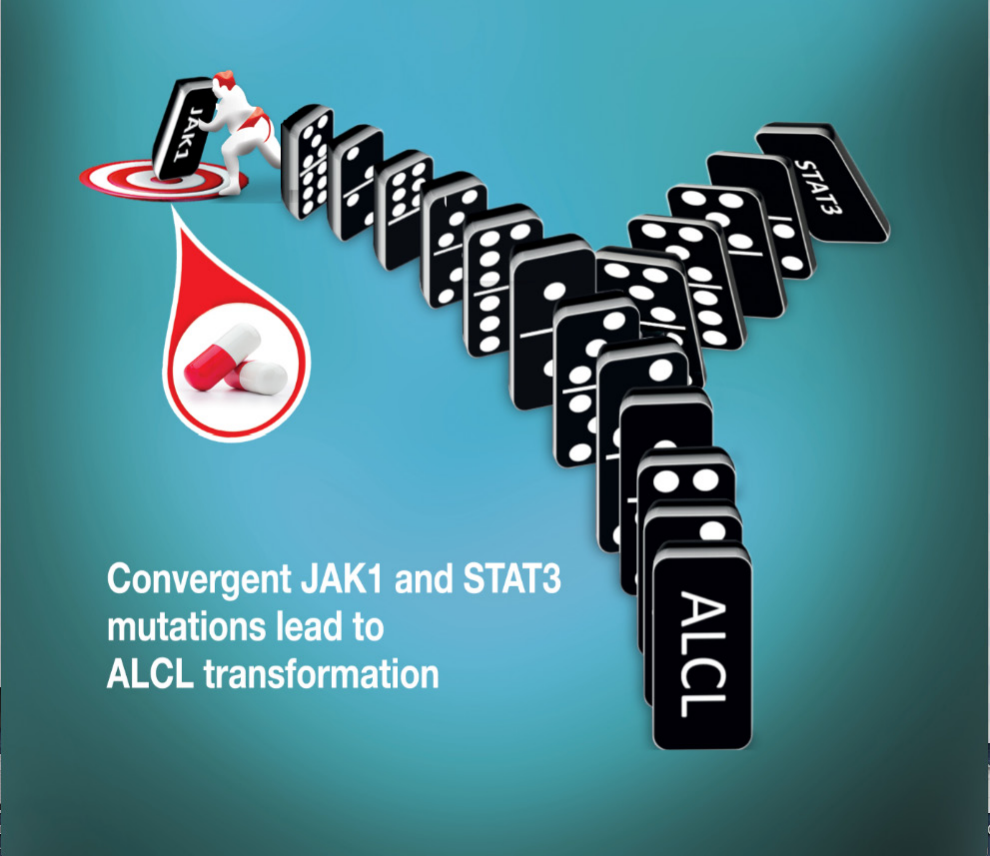
Convergent effect of JAK1 and STAT3 mutations in ALK - Anaplastic Large Cell Lymphoma (ALCL) Activating mutations are most oncogenic when they are concomitantly expressed. However, the inhibition of JAK1 enzymatic activity by selective small molecules impairs STAT3 activation and transformation. The domino chips represent the signaling cascade and the convergent effects fostering the maintenance of the neoplastic phenotype of ALCL. The sumo fighter signifies the therapeutic capacity of target inhibitors blocking JAK1 activation. Additional chips indicate other known and unknown events. Cover designed with the assistance of FenicePool.

Although JAK1/STAT3 mutants are oncogenic, they require lymphokine engagement (i.e. IL-6, IL-23) and JAK activation, whose inhibition (Ruxolitinib, HSP90) leads to STAT3 dephosphorylation, cell growth inhibition and lymphoma control in a Patient Derived Tumor Xenografted (PDTX) model. In addition, STAT3 hyperactivation forces the preferential differentiation of T-cells and the secretion of selective lymphokines (IL-17, IL-22). Therefore, the inflammatory phenotype associated with ALCL hijacks host elements and favors protumorigenic phenotypes (i.e. neutrophils, macrophages) and/or the emergency recruitment of immunosuppressive T-cells.

These data validate JAK and STAT as therapeutic targets. Inhibitors of JAKs were recently introduced and it is anticipated that these new therapies, either alone or in combination, might be beneficial [7-8]. We predict these compounds, which generally have reasonable toxicities, might limit the adverse side effects of conventional chemotherapy and/or allow lower doses, possibly increasing responses and sustained remissions. There are several compounds that are currently being tested or have been approved for different disorders that could be evaluated in molecularly stratified cancer patients. Thus, the genotyping of future lymphoma patients will become invaluable to the treatment of disease. Our groups are now pursuing this strategy, hoping to identify naïve and/or refractory patients, who are eligible for novel targeted therapies. These studies are critical to determine the oncogenic role of the JAK/STAT pathways along different clinical stages. This, in combination with the execution of co-clinical trials and Patient Derived Tumor Xenograft (PDTX) models, should open new personalized/precision therapies that will undoubtedly improve clinical success and patient outcomes.

## References

[1] Stark G.R (2012). Immunity.

[2] Scott L. M (2015). Blood Rev.

[3] Yu H (2014). Nat Rev. Cancer.

[4] Barreca A (2011).

[5] Chiarle R (2005). Nat Med.

[6] Crescenzo R (2015). Cancer Cell.

[7] Mascarenhas J.O (2014). Blood Rev.

[8] Buchert M (2015). Oncogene.

